# The application of enzymatic fermented soybean effectively regulates associated microbial communities in tea soil and positively affects lipid metabolites in tea new shoots

**DOI:** 10.3389/fmicb.2022.992823

**Published:** 2022-08-23

**Authors:** Shuning Zhang, Litao Sun, Yujie Shi, Yujie Song, Yu Wang, Kai Fan, Rui Zong, Yusheng Li, Linjun Wang, Caihong Bi, Zhaotang Ding

**Affiliations:** ^1^Tea Research Institute, Qingdao Agricultural University, Qingdao, China; ^2^Tea Research Institute, Shandong Academy of Agricultural Sciences, Jinan, China; ^3^Qingdao Hexie Biotechnology Co., Ltd., Qingdao, China; ^4^Shandong Agricultural Technology Extension Center, Jinan, China; ^5^Weihai Agricultural and Rural Affairs Service Center, Weihai, China; ^6^Linyi Agricultural Technology Extension Center, Linyi, China

**Keywords:** tea, fermented soybean, fertilization, microbial communities, metabolite profiling

## Abstract

Compared with traditional organic fertilizer, fermented soybean is a better fertilizer resource in tea plantations. The application of organic fertilizer is a feasible practice to mitigate the soil degradation caused by the overuse of chemical fertilizers, which can effectively regulate soil microbial communities in tea plantations. However, the effects of fermented soybean on soil microbial communities, soil metabolites and metabolites in tea new shoots have not been systematically demonstrated, and their interactions have never been studied. Here, we investigated the responses of the soil microbial community, soil metabolites and metabolites of tea new shoots to urea fertilization (UF), naturally fermented soybean fertilization (NFS) and enzymatic fermented soybean fertilization (EFS), and analyzed the relationships between soil microbes, soil metabolites and metabolites in tea new shoots. The results showed that soil bacterial communities were dominated by *Pseudomonas, Romboutsia, Candidatus_Nitrosotalea* and *Helicobacter*, and soil fungal communities were dominated by *Peziza, Fusarium, Candida* and *Cheilymenia* at the genus level. In EFS, bacterial genera (*Glutamicibacter* and *Streptomyces*) and fungal genera (*Candida* and *Actinomucor*) presented high abundances, which were correlated with soil carbohydrate and lipid including D-Mannitol, D-Sorbitol, 9,12-Octadecadienoic acid and (Z)-13-Docosenoic acid. Enzymatic fermented soybean fertilization also affected the lipid metabolites in tea new shoots. Glycerolipids and glycerophospholipids significantly increased in EFS, which positively correlated with some soil microbial communities. Besides, the application of fermented soybean fertilizer could increase the contents of TP, AP and AK, which were also important environmental factors affecting the structure of soil microbial community in tea plantation. It was concluded that fermented soybean fertilization could improve soil nutrition, regulate associated microbial communities, and positively affect lipid metabolites in tea new shoots. This study not only explores the relationships between soil microbes and metabolites in tea plants, but also provides feasible technical guidance to cultivate high-quality tea using soybean as high-grade fertilizer.

## Graphical Abstract

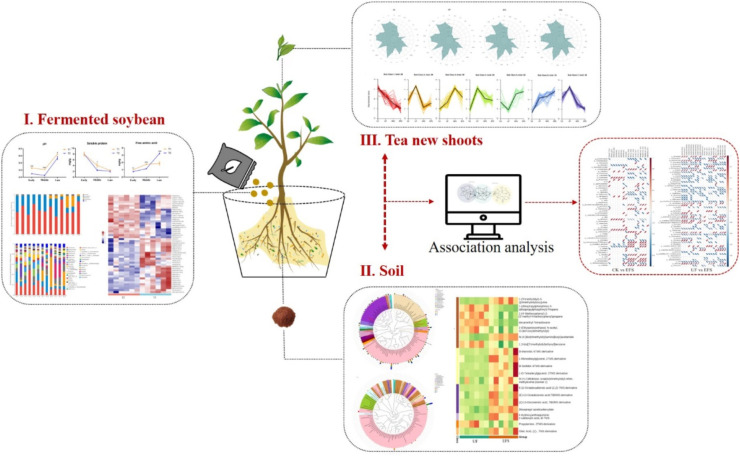

## Introduction

Tea (*Camellia sinensis* (L.) Kuntze) is a perennial evergreen leaf-used economic plant. Its internal rich metabolites confer tea elegant flavors and healthy function (Wachira et al., 2013). The tea taste was influenced by well-characterized non-volatile metabolites including polyphenols, flavonoids, theanine and alkaloids, and the source of tea aroma was volatile metabolites including terpenoids, phenylpropanoids and fatty acid derivatives (Yang et al., 2013; Daglia et al., 2014). Among them, lipid was the main contributor to the generation of flavor and aroma compounds in tea leaves. The oxidation products of free fatty acids including (Z)-3-hexenol, its esters and (E)-2-hexenal principally contribute to the fresh odor of green tea (Ho et al., 2015). The changes of lipids in tea plants could affect the formation of aroma-generating compounds and thus the final tea quality (Liu et al., 2017). In addition, plants and their microbiome were highly interlinked and might have co-evolved to function as integrated ecologies (Pang et al., 2021). Soil microbiomes, as the second genome of plants, could provide plants with microbe-derived metabolites and traits (Pascale et al., 2020). Some microbiomes could assist the plant in nutrient absorption, pathogen resistance and growth promotion (Turner et al., 2013). Kudjordjie et al. (2019) and Maggini et al. (2020) reported that the key microbiomes played important roles in promoting the growth and development of maize and echinacea plants. Korenblum et al. (2020) investigated that rhizosphere bacteria could trigger the systemic exudation of acyl sugars and changed the leaf metabolites and transcriptomes in tomato plants (Korenblum et al., 2020). In tea plants, the tea microbiome has shown that it could help the plant with soil nutrient acquisition and stress management. Arbuscular Mycorrhizal Fungi (AMF) and some beneficial microorganisms could colonize in tea roots, which was beneficial to tea growth by increasing the contents of amino acids, protein, caffeine, and polyphenols (Bag et al., 2022).

Soil is a complex bioreactor and a nutrition stronghold of microbial communities. Thousands of bacteria and fungi coexisted in soil using various organic carbon sources (Fierer, 2017). Soil is also the main hub of fertilizer transportation and can act directly on plant roots (Edwards et al., 2015; Bonanomi et al., 2018). In recent years, tea industry had increasingly moved toward refinement, modernization and intensification. More and more tea farmers applied heavy chemical fertilization for high yield and quality components in tea plantations, which could cause environment problems and soil degradation (Ni et al., 2018; Yang et al., 2018). It could finally cause inhibit tea growth and affect the tea quality and yield (Fung et al., 2008; Arafat et al., 2019). So, the organic fertilizer was applied as direct and effective management in tea plantations to realize the synchronization of nutrient supply and quality improvement of tea plants. Soybean was an excellent choice with rich in high-quality plant protein, which was usually used as organic fertilizer in tea plantations of China. In agricultural production, soybeans should need a solid fermentation process before application, which could improve the effective utilization. However, it might lead to some problems such as malodorous gas volatilization and inadequate conversion (Gupta et al., 2018). Currently, the addition of enzymes and bacteria could accelerate the fermentation processes and reduce the risk of fermentation failure (Park et al., 2010). Lee et al. (2014) and Park and Kim (2020) reported that the effects of various microbial starters on volatile and non-volatile metabolites during soybean fermentation, indicating biotransformation in fermentation had the potential to improve the soybean nutritional value. Therefore, enzymes and bacteria were applied in soybean fermentation to obtain better fertilizer effects.

In recent years, the direct analyses of microbial communities using high-throughput sequencing technology have become more and more accurate and effective. The combination of microbiome research and multi-omics methods has deepened our understanding of the relationships between microbiomes and plants. However, it is far from enough to pay attention to the single effect of soil nutrition and tea quality improvement after organic fertilization in tea plantation, which seriously ignores the relationships between the underground and aboveground parts of tea plants. The interactions of soil microbiomes, soil metabolites and metabolites in tea new shoots remain unclear. In this study, we investigated microbial communities and metabolites in soybean in the naturally fermented way and enzymatic fermented way. After fertilization, we studied the effects of naturally fermented soybean fertilization and enzymatic fermented soybean fertilization on soil microbial communities, soil metabolites and metabolites of tea new shoots by using 16S and ITS rRNA sequencing, GC-MS, and UPLC-MS, respectively. We also analyzed the relationships of key microbial communities, key metabolites in soil and metabolites of tea new shoots. This study provided feasible technical guidance for fermented soybean application in tea plantation. In addition, exploring the microbial communities related to the metabolites of tea new shoots can also have potential contributions to tea flavor improvement.

## Materials and methods

### Soybean fermentation and collection

The experiment was conducted in Qingdao, Shandong Province, China (36° 18’ N, 120° 7’ E) in 2020. Fermented soybeans were prepared in plastic barrels with 50 L. The diameter of the barrel bottom is 38 cm, and the height is 58 cm. Before fermentation, each barrel needs to be cleaned and air dried. In this experiment, we used natural fermentation (T1) and enzyme fermentation (T2) to ferment soybean. Firstly, 5 kg soybeans and 9 L sterile water were added to each fermentation barrel. Soybean and water were fully mixed in the ratio of 1:1.8. The enzyme preparation was a mixed powder with bacteria and enzymes (the number of effective viable bacteria ≥ 20 billion/g and the total enzyme activity > 10,000 u/g). The main bacteria were *Saccharomycetes, Lactobacillus, Brevibacterium* and *Bacillus licheniformis*, and the enzymes mainly included cellulase, lipase and neutral xylanase. This was added to the soybean at a 2‰ ratio based on the total weight of soybeans and water. The whole fermentation process was facultative anaerobic fermentation, which lasted for 21 days. At 3, 12, and 21 days, fermented soybeans were collected at random under aseptic conditions. Then, each sample with six replications was divided into two parts, one part was stored at 4°C to determine its physical and chemical properties, and the other part was frozen at −80°C to determine its bacterial composition and metabolites.

### Fermented soybean sampling and analysis

Microbial DNA was extracted from fermented soybean using the E.Z.N.A.^®^ soil DNA Kit (Omega Bio-Tek, Norcross, GA, United States). The final DNA concentration and purification were determined by NanoDrop 2000 UV–vis spectrophotometer (Thermo Scientific, Wilmington, NC, United States), and DNA quality was checked by 1% agarose gel electrophoresis. The V3–V4 hypervariable regions of the bacterial 16S rRNA gene were amplified with primers 338Fand 806R by thermocycler PCR system (GeneAmp 9700, ABI, Foster City, CA, United States). PCR amplification and Illumina MiSeq sequencing were performed according to previous methods (Zhang et al., 2020c).

UHPLC-MS analysis of fermented soybean was performed on a Thermo UHPLC system equipped with an ACQUITY BEH C18 column (100 mm × 2.1 mm i.d, 1.7 μm; Waters, Milford, MA, United States). Firstly, the metabolites were extracted using a 400 μl methanol: water (4:1, v/v) solution. The mixture was allowed to settle at −20°C and treated by a high throughput tissue crusher Wonbio-96c (Shanghai Wanbo Biotechnology Co., Ltd., Shanghai, China). The samples were placed and centrifugated, and the supernatant was carefully transferred to sample vials for LC-MS/MS analysis. The mobile phases consisted of 0.1% formic acid in water (solvent A) and 0.1% formic acid in acetonitrile: isopropanol (1:1, v/v; solvent B). Then, the mass spectrometric data were collected using a Thermo UHPLiC-Q Exactive Mass Spectrometer equipped with an electrospray ionization (ESI) source operating in either positive or negative ion mode. After UPLC-TOF/MS analyses, the raw data were imported into the Progenesis QI 2.3 (Nonlinear Dynamics, Waters, Milford, MA, United States) for peak detection and alignment. Finally, quality control and data annotation were performed.

### Pot experiment

The experiment was conducted in Qingdao, Shandong Province, China (36°18′N, 120°7′E) in 2020. A 2-year-old tea seeding of “*Zhongcha 108*” [*C. sinensis* (L.) O. Kuntze] were planted in plastic pots with 28 cm diameter and 18 cm high. Four tea plants were in each pot. The soil was brown loam with a pH of 5.92, soil organic matter of 36.92 g/kg and total nitrogen content of 1.13 g/kg. The environmental conditions in the greenhouse were as followed: the temperature was 26°C/20°C (day/night), air humidity was 55% and light time lasted 12 h per day. The experiment consisted of four treatments: control (CK), urea fertilization (UF), naturally fermented soybean fertilization (NFS) and enzymatic soybean fertilization (EFS). Each treatment consisted of six replicates. The application of nitrogen content was based on the previous study (Yang et al., 2018). 3 g urea was applied to each pot in UF, and 20 g fermented soybean was applied to each pot in NFS and EFS. The fertilizer position was 5 cm on the side of each tea seedling and 10 cm in depth. Finally, the fertilizer holes were covered with topsoil and watered adequately. One month after fertilization, the soil and tea new shoots were sampled 1 month after fertilization. Soil samples were divided into two parts: one part was frozen quickly in liquid nitrogen and stored at −80°C until the microbial communities and soil metabolites were analyzed, and the other part was dried naturally in the room and then analyzed for physicochemical indexes. Tea new shoots were frozen quickly in liquid nitrogen and stored at -80°C until metabolites were analyzed.

### Soil sampling and analysis

Microbial DNA was extracted from soil using the E.Z.N.A.^®^ soil DNA Kit (Omega Bio-Tek, Norcross, GA, United States). DNA concentration was detected by agarose gel electrophoresis PCR amplification was performed. The V4 hypervariable regions of the bacterial 16S rRNA gene were amplified with primers 515F and 806R by thermocycler PCR system (GeneAmp 9700, ABI, United States), and the ITS1 hypervariable regions of the fungal ITS gene were amplified with primers ITS5-1737F and ITS2-2043R. The purified amplicons were pooled and constructed using TruSeq^®^ DNA PCR-Free Sample Preparation Kit library construction Kit. The constructed libraries were quantified by Qubit and Q-PCR and then sequenced by NovaseQ6000.

GC-MS analysis of soil was performed on Agilent 7890B gas chromatograph coupled to a 7000D mass spectrometer with a DB-5MS column (30 m length × 0.25 mm i.d. × 0.25 μm film thickness, J&W Scientific, Folsom, CA, United States). Firstly, the freeze-dried soil materials were crushed using a mixer mill (MM 400, Retsch) with a zirconia bead for 1.5 min at 30 Hz. 500 mg powder was diluted to a 500 μl with methanol: isopropanol: water (3:3:2 V/V/V), vortexed for 3 min and ultrasound for 30 min. The extracts were centrifuged at 12,000 r/min under 4°C for 3 min. Then, helium was used as the carrier gas, at a flow rate of 1 ml/min. Injections were made in the front inlet mode with a split ratio of 10:1 and the injection volume was 1 μL. The oven temperature was held at 40°C for 1 min and then raised to 100°C at 20°C/min, raised to 300°C at 10°C/min, and held at 300°C for 5 min. All samples were analyzed in scan mode. The injector inlet and transfer line temperatures were 250 and 280°C, respectively. Finally, quality control and data annotation were performed.

### Tea new shoots sampling and analysis

The extracts and determination of each sample were performed by Wuhan Metware Biotechnology Co., Ltd., China^1^ using a UPLC-MS/MS system (UPLC, SHIMADZUNexera X2; MS, Applied Biosystems 4500 Q TRAP) equipped with an Agilent SB-C18 (1.8 μm, 2.1 mm × 100 mm). Firstly, tea new shoots were freeze-dried by the vacuum freeze-dryer and crushed by a mixer mill. 100 mg of lyophilized powder were dissolved with 1.2 ml 70% methanol solution and placed in a refrigerator at 4°C overnight. Following centrifugation at 12,000 rpm for 10 min, the extracts were filtrated (SCAA-104, 0.22 μm pore size; ANPEL, Shanghai, China^2^) before UPLC-MS/MS analysis. The mobile phase consisted of solvent A, pure water with 0.1% formic acid, and solvent B, acetonitrile with 0.1% formic acid. Sample measurements were performed with a gradient program that employed the starting conditions of 95% A and 5% B. Within 9 min, a linear gradient to 5% A, 95% B was programmed, and a composition of 5% A, 95% B was kept for 1 min. Subsequently, a composition of 95% A and 5.0% B was adjusted within 1.10 min and kept for 2.9 min. The flow velocity was set as 0.35 ml per minute; The column oven was set to 40°C; The injection volume was 4 μl. The effluent was alternatively connected to an ESI-triple quadrupole-linear ion trap (QTRAP)-MS. Quality control and data annotation were performed according to previous methods.

### Statistical analysis

One-way analysis of variance (ANOVA) with Duncan’s test was used to determine significant differences (*p* < 0.05) among soybean nutrition analysis, soil properties and enzyme activities, as well as the relative abundance of microbial taxa. The LDA Effect Size algorithm used the non-parametric factorial Kruskal Wallis sum rank test to detect microbial groups with significant abundance differences. The Circos graph was used to visualize the composition of microbial communities using Circos-0.67-7 software.^3^ The HCA (hierarchical cluster analysis) results of samples and metabolites were presented as heatmaps with dendrograms, while Pearson correlation coefficients (PCC) between samples were calculated by the cor function in R and presented as only heatmaps. Both HCA and PCC were carried out by R package. For HCA, normalized signal intensities of metabolites (unit variance scaling) are visualized as a color spectrum. Significantly regulated metabolites between groups were determined by VIP and absolute Log_2_FC (fold change). VIP values were extracted from the OPLS-DA result containing score plots and permutation plots were generated using the R package MetaboAnalyst. The data was log transform (log_2_) and mean centering before OPLS-DA. To avoid overfitting, a permutation test (200 permutations) was performed. The heatmaps about metabolites were normalized by unit variance scaling and processed “pheatmap” in R software, and the heatmaps about the correlation between differential metabolites and microorganisms were normalized by unit variance scaling and processed “corrplot” in R software. All statistical analyses were performed using the R platform (version3.5.0).

## Results

### Effects of different fermentation methods on soybean fermentation

To evaluate the effects of different fermentation ways on soybean, we analyzed the pH value and nutrition contents at the early, middle and late stages of fermentation. In the whole fermentation process, the pH value of T2 was significantly lower than that of T1. As the fermentation process went on, the content of soluble protein in soybean gradually decreased, while the content of free amino acids gradually increased. The content of free amino acids in T2 was significantly higher than that in T1 at the late stage of fermentation (Supplementary Figure 1A). In addition, we also analyzed the contents of amino acids in fermented soybean. In two fermentation ways, the contents of tyrosine and histidine were relatively higher, and the contents of cystine and serine were lower than the other amino acids. The contents of 20 amino acids in T2 were significantly higher than that in T1 (Supplementary Figure 1B). At the last stage of fermentation, the nutrients in soybean were fully decomposed. So, the following analysis was focused on soybean in the late stage of fermentation.

To analyze the changes of bacterial communities in fermented soybeans, we studied the composition and structure of bacterial communities at the phylum and genus level using 16S rRNA gene sequencing. At the phylum level, *Firmicutes, Proteobacteria, Bacteroidota* and *Actinobacteriota* were the dominant phyla in fermented soybean (Supplementary Figure 2A). At the genus level, *Lactobacillus, Ignatzschineria, Peptoniphilus, Enterococcus* and *Bacteroides* were the dominant genus in fermented soybean (Supplementary Figure 2B). In addition, we also studied the key bacterial communities that could explain the differences between natural fermentation and enzymatic fermentation using linear discriminant analysis of effect size (LEfSe; Supplementary Figure 2C). Overall, the bacterial communities with significant differences in T2 were different from that in T1. Specifically, significant enrichments of *Proteobacteria, Firmicutes* and *Bacteroidota* were in T2. *Proteobacteria* included *g_Ralstonia, g_norank_f_norank_o_Enterobacterales, o_Sphingomonadales, o_Rhizobiales* and *c_Alphaproteobacteria*. *Firmicutes* included *g_Peptoniphilus, g_Gallicola, g_Bacillus, g_Erysipelothrix* and *g_Leuconostoc*. *Bacteroidota* included *g_Bacteroides, f_Bacteroidaceae, g_Dysgonomonas* and *g_Sphingobacterium*. Therefore, the species of bacterial community with high abundances did not change significantly, but the internal structure of the bacterial community changed significantly in soybean with enzymatic fermentation.

To reveal the marked differences of metabolites in fermented soybeans, lipid metabolites were performed. A total of 247 lipid molecular species were identified in fermented soybean. The Euclidean distance coefficient method was used to visualize the lipid metabolites with significant differences between natural fermentation and enzymatic fermentation in soybeans (Supplementary Figure 3A). According to the selecting criteria of fold change, VIP value and *p*-value (fold change ≥ 1.5 and fold change ≤ 0.67; VIP ≥ 1; *p* < 0.05), 22 lipid metabolites exhibited significant up-regulation in EFS including glycerophospholipids [PA(18:0/16:0), PA(24:0/18:2), PA(16:0/18:3), LPMe(14:0), PMe(16:0/18:3), PMe(18:2/23:0) and PG(18:3e/18:1)], glycerolipids [DGMG(18:2), MGDG(16:0/12:2), MGDG(18:1/29:2), MGDG(18:2/10:0), MG(18:3), TG(27:0/18:0/18:2) and DG(15:0/18:1)], and sphingolipid [Cer(m17:1/18:2), Cer(d17:1/17:0 + O), Cer(d18:1/18:2 + 2O), Cer(t18:1/18:0 + 2O), Cer(t18:1/18:0 + 2O), Cer(t17:1/16:0 + O), CerG2GNAc1(m19:1/18:3) and Hex2Cer(d23:0/18:2)]. To explore the correlations between metabolites and bacterial communities in fermented soybeans, we used spearman coefficient to calculate the correlation coefficient and determined the statistically significant correlation through rank correlation test (Supplementary Figure 3B). The relative abundances of *Bacillus, Bacteroides, Gallicola, Leuconostoc* and *Neoscardovia* were positively correlated with ceramides, while the relative abundances of *Acinetobacter, Cronobacter, Enterobacter, Rummeliibacillus* and *Microvirgula* were negatively correlated with ceramides. Glycerolipid metabolites [DG(18:1/23:0), DG(18:1/24:0), DG(19:1/18:1), DG(16:0e/20:1), TG(4:0/18:1/18:2), TG(16:0/6:0/18:2) and TG(20:1/18:2/18:3)] showed opposite correlation with ceramide metabolites.

### Effects of fermented soybean on soil environmental factors and microbial communities

To evaluate the soil physicochemical indexes after fermented soybean fertilization, we analyzed the nutrient contents and enzyme activities in tea soils (Tables 1, 2). Compared with CK, soil pH values in UF, NFS and EFS significantly reduced, but the pH value was the lowest in UF, at 5.19. The contents of TP, AP and AK significantly increased in soils after fermented soybean fertilization, especially in enzymatic fermented soybean fertilization. In addition, soybean fertilization stimulated the activities of some enzymes, including solid-sucrase (S_SC), solid-acid phosphatase (S_ACP), solid-urease (S_UE) and solid-acid protease (S_ACPT).

**TABLE 1:** The physicochemical properties of soils after fermented soybean fertilization.

	pH	OM g/kg	TN g/kg	TP g/kg	TK g/kg	AN mg/kg	AP mg/kg	AK mg/kg	Ca g/kg	Mg g/kg	Na g/kg	Cu mg/kg	Zn mg/kg
CK	6.68 ± 0.11a	93.77 ± 35.13b	2.55 ± 0.40c	0.64 ± 0.20b	14.03 ± 1.03a	70.16 ± 7.99c	131.20 ± 34.34b	285.53 ± 13.62b	3.86 ± 0.37a	2.68 ± 0.03ab	1.64 ± 0.24a	18.22 ± 8.07a	83.62 ± 6.41a
UF	5.19 ± 0.16c	175.52 ± 82.59ab	4.76 ± 1.71ab	0.82 ± 0.18b	11.26 ± 2.01a	186.50 ± 8.16a	158.77 ± 48.40b	371.37 ± 92.03b	3.85 ± 0.40a	2.42 ± 0.12ab	1.60 ± 0.06a	10.81 ± 3.09a	74.88 ± 4.38ab
NFS	6.21 ± 0.14b	192.29 ± 27.38ab	5.97 ± 2.20a	0.93 ± 0.11ab	8.39 ± 4.98a	146.64 ± 11.10b	245.17 ± 33.21ab	436.27 ± 45.00ab	3.71 ± 0.40a	2.29 ± 0.42c	1.87 ± 0.76a	12.08 ± 3.30a	72.33 ± 3.96b
EFS	6.31 ± 0.14b	209.37 ± 47.40a	4.91 ± 1.22ab	1.25 ± 0.18a	11.69 ± 0.99a	125.46 ± 13.37b	410.42 ± 147.52a	695.27 ± 176.77a	3.60 ± 0.20a	2.84 ± 0.30a	1.88 ± 0.89a	14.43 ± 5.37a	78.10 ± 4.16ab

Values with the same letter are not significantly different (*p* < 0.05).

CK, control experiment; UF, urea fertilization; NFS, naturally fermented soybean fertilization; EFS, enzymatic fermented soybean fertilization.

**TABLE 2:** The activities of some enzymes in soils after fermented soybean fertilization.

	S-ACP (nmol/h/g)	S-UE (μ g/d/g)	S-CAT (μ mol/h/g)	S-CL (μ g/d/g)	S-ACPT (μ g/h/g)	S-SC (mg/d/g)
CK	1387.62 ± 313.27b	783.83 ± 52.79c	739.73 ± 52.07ab	303.78 ± 32.17a	16.45 ± 8.29b	29.85 ± 1.11c
UF	1073.41 ± 45.67b	1017.27 ± 107.68bc	744.50 ± 62.76ab	322.42 ± 47.68a	32.90 ± 14.15ab	29.62 ± 2.06c
NFS	2687.83 ± 92.49a	1258.29 ± 149.95ab	687.37 ± 81.87b	344.58 ± 107.54a	63.45 ± 19.70a	56.24 ± 6.74a
EFS	2718.61 ± 733.84a	1418.29 ± 124.51a	821.88 ± 42.32a	488.32 ± 245.45a	60.67 ± 8.23a	45.77 ± 3.88b

Values with the same letter are not significantly different (*p* < 0.05).

CK, control experiment; UF, urea fertilization; NFS, naturally fermented soybean fertilization; EFS, enzymatic fermented soybean fertilization.

**FIGURE 1:**
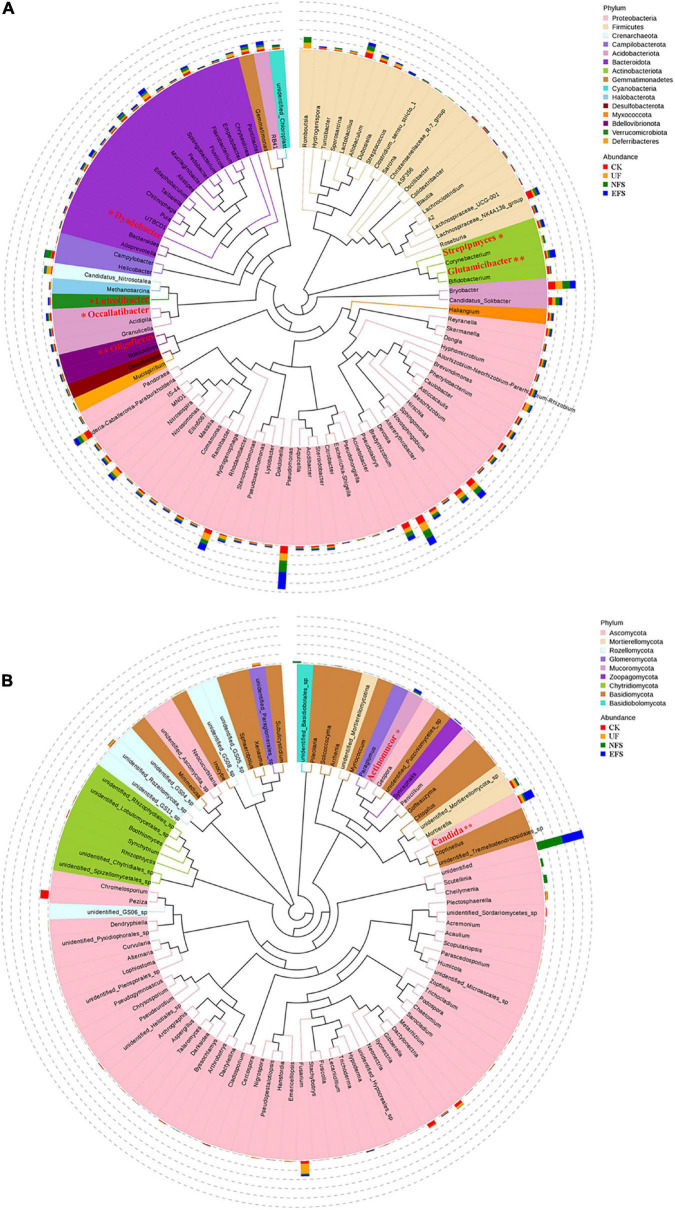
The composition of microbial communities in soil with fermented soybean fertilization. **(A)** The phylogenetic tree of soil bacterial communities. **(B)** The phylogenetic tree of soil fungal communities.

To analyze the microbial composition in the soils after fermented soybean fertilization, we aligned the dominant OTUs with the Silva 119 and Unite database at the phylum and genus levels (Figure 1). In bacterial communities, the dominant phyla were *Proteobacteria, Bacteroidota, Firmicutes, Acidobacteriota* and *Actinobacteriota*. The dominant genera were *Pseudomonas, Romboutsia, Candidatus_Nitrosotalea, Helicobacter, Bryobacter* and *Bradyrhizobium* (Figure 1A). In fungal communities, the dominant phyla were *Ascomycota, Mortierellomycota, Rozellomycota, Glomeromycot* and *Mucoromycota*. The dominant genera were *Peziza, Fusarium, Candida, Cheilymenia, Mortierella* and *Dactylonectria* (Figure 1B). To further confirm the differences of microbial communities at the genus level, we used the *t*-test test to select microbial communities with significant differences in soils. Microbial communities with significant differences were shown in red color (Figure 1). In bacterial communities, *Glutamicibacter, Oligoflexus, Luteolibacter, Dyadobacter, Streptomyces* and *Occallatibacter* presented high abundances in EFS. In fungal communities, *Candida* and *Actinomucor* presented high abundances in EFS.

**FIGURE 2:**
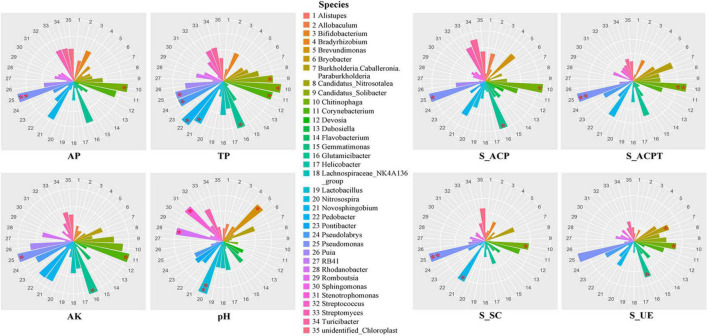
The rose diagrams of relationships between soil key properties and bacterial communities in soils with fermented soybean fertilization.

To explore the influences of soil environmental factors on soil bacterial communities, we analyzed the relationships between dominant genus and soil properties using spearman correlation (Figure 2). In terms of soil properties, AP, AK, TP and pH were important environmental factors in soils. The results showed that the soil AP was significantly related to the relative abundances of *Pseudomonas* and *Chitinophaga*. The soil AK was significantly related to the relative abundances of *Pseudomonas, Glutamicibacter* and *Croynebacterium*. The soil TP was significantly related to the relative abundances of *Pseudomonas, Pseudolabrys, Pedobacter, Novosphingobium, Glutamicibacter, Chitinophaga, Croynebacterium* and *Candidatus_solibacter.* The soil pH was significantly related to the relative abundances of *Nitrosospira, Brevundimonas, Rhodanobacter and Stenotrophomonas.* In terms of soil enzymic activities, S_ACP, S_ACPT, S_SC and S_UE were important environmental factors in soils. The results showed that the S_ACP, S_ACPT and S_UE were strongly associated with *Chitinophaga, Glutamicibacter* and *Pseudomonas.* The S_SC was strongly associated with *Chitinophaga, Novosphingobium and Pseudomonas.*

### Effects of fermented soybean on soil metabolites

**FIGURE 3:**
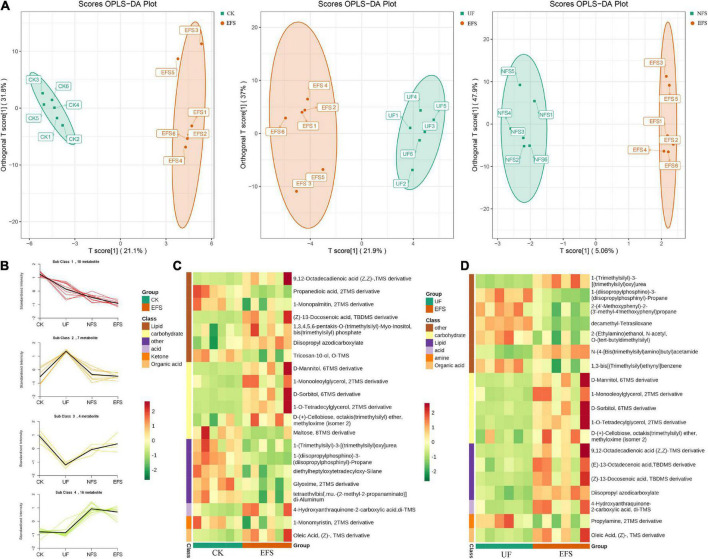
The differential metabolites of soil with fermented soybean fertilization. **(A)** The OPLS-DA analysis of soil metabolites. **(B)** The K-means cluster analysis of lipid molecules in tea soils. **(C)** The heatmap of soil differential metabolites in CK vs EFS group. **(D)** The heatmap of soil differential metabolites in UF vs EFS group.

**FIGURE 4:**
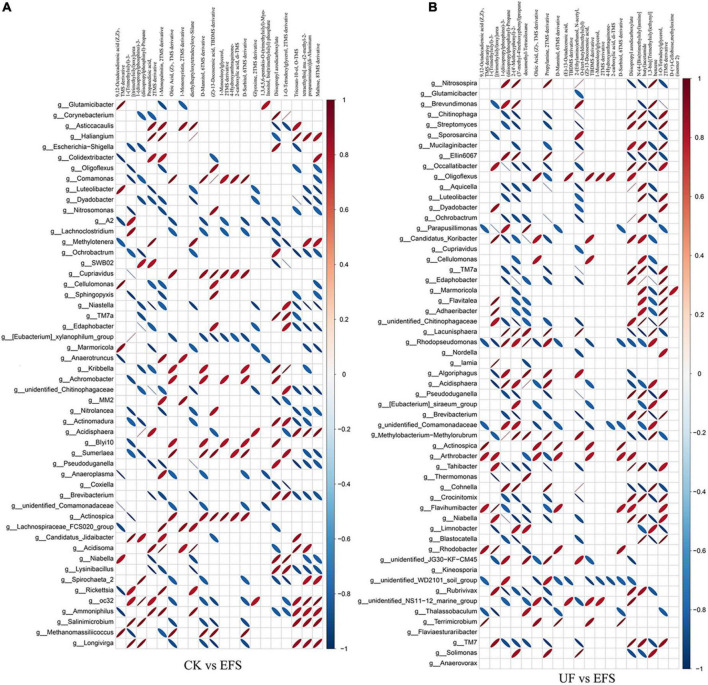
The ellipse heatmap of relationships between soil bacterial communities and soil metabolites. **(A)** The relationship of soil bacterial communities and soil metabolites in CK vs EFS group. **(B)** The relationship of soil bacterial communities and soil metabolites in UF vs EFS group.

**FIGURE 5:**
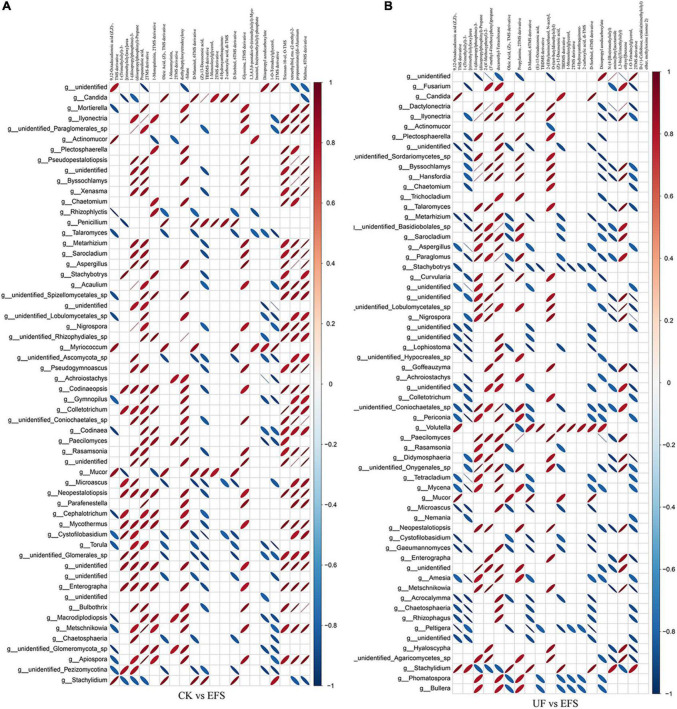
The ellipse heatmap of relationships between soil fungal communities and soil metabolites. **(A)** The relationship of soil fungal communities and soil metabolites in CK vs EFS group. **(B)** The relationship of soil fungal communities and soil metabolites in UF vs EFS group.

To elucidate the metabolic changes in soils after fermented soybean fertilization, we detected and screened out metabolites with significant differences in soils. The S-plot of the OPLS-DA model was used to maximize the discrimination among EFS treatment and other treatments, including CK vs EFS, UF vs EFS and NFS vs EFS (Figure 3A). The results showed that two treatments of these three contrast groups were located, respectively, on the positive and negative sides of the x-axis, indicating that the OPLS-DA model was a goodness of fit as a predictive model to evaluate the variation in metabolite profiles. Firstly, soil metabolites with different fertilizations were distributed into 4 clusters by k-means cluster analysis (Figure 3B). The contents of metabolites in cluster 4 of NFS and EFS were higher than in CK and UF. In cluster 4, 16 metabolites were found including lipid, carbohydrate, organic acid and amino acid. Soil metabolites with significant differences were further screened out. In total, 106 metabolites were detected in soils. Compared with CK, there were 21 metabolites with significant differences in EFS (Figure 3C). Therein, lipid, carbohydrate and acid substances were significantly up-regulated including (Z)-13-Docosenoic acid, 9,12-Octadecadienoic acid, D-Mannitol, D-Sorbitol, 1-O-Tetradecylglycerol, 1-Monooleoylglycerol and oleic acid, etc. Compared with UF, 19 metabolites with significant differences in EFS (Figure 3D). Therein, lipid, carbohydrate and acid substances were significantly up-regulated, including (Z)-13-Docosenoic acid, 9,12-Octadecadienoic acid, D-Mannitol, D-Sorbitol and oleic acid, etc. Compared with NFS, there were no metabolites with significant differences in EFS. In addition, we analyzed the correlations between microbial communities with significant differences and metabolites in soil (Figures 4, 5). Compared with CK, carbohydrate, organic acid and lipid substances established close relationships with soil bacterial communities in EFS (Figure 4A). *Comamonas, Cupriavidus, Kribbella, Achromobacter, Sumerlaea* and *Methanomassiliicoccus* were positively correlated with carbohydrate and oleic acid including D-Mannitol, D-Sorbitol and oleic acid. *Cellulomonas* and *Methanomassiliicoccus* were positively correlated with lipid including 9,12-Octadecadienoic acid (Z, Z)- and (Z)-13-Docosenoic acid. Compared with NFS, carbohydrate and lipid substances also established close relationships in EFS (Figure 4B). *Actinospica, Arthrobacter, Flavihumibacter, Rhodobacter* and *Terrimicrobium* were positively correlated with carbohydrates, while *Parapusillimonas, Rhodopseudomonas* and *Thalassobaculum* were negatively correlated with carbohydrates including D-Mannitol and D-Sorbitol. *Actinospica, Arthrobacter* and *Terrimicrobium* were positively correlated with lipid, while *Rhodopseudomonas* was negatively correlated with lipid including 9,12-Octadecadienoic acid (Z, Z)-, (Z)-13-Docosenoic acid and Diisopropyl azodicarboxylate. In fungal communities, compared with CK, carbohydrate and lipid substances established close relationships with fungal communities in EFS (Figure 5A). *Candida* was positively correlated with (Z)-13-Docosenoic acid. *Mucor* and *Stachylidium* were positively correlated with 9,12-Octadecadienoic acid (Z, Z)- and (Z)-13-Docosenoic acid, while *Cystofilobasidium* and *Cephalotrichum* were negatively correlated with these two lipid metabolites. *Candida, Penicillium, Myriococcum, Mucor* and *Stachylidium* were positively correlated with D-Mannitol and D-Sorbitol, while *Rhizophlyctis, Talaromyces, Microascus, Cystofilobasidium, Torula*, and *Chaetosphaeria* were negatively correlated with these two carbohydrate metabolites. Compared with UF, carbohydrate and lipid substances established close relationships with soil fungal communities in EFS (Figure 5B). *Volutella, Mucor* and *Stachylidium* were positively correlated with 9,12-Octadecadienoic acid (Z, Z)- and (Z)-13-Docosenoic acid, while *Metarhizium, Stachybotrys, Lophiostoma, Microascus, Cystofilobasidium, Gaeumannomyces, Acrocalymma* and *Peltigera* were negatively correlated with these lipid metabolites. *Volutella* and *Mucor* were positively correlated with D-Mannitol and D-Sorbitol, while *Stachybotrys, Peltigeta, Rhizophagus, Chaetosphaeria, Acrocalymma* and *Gaeumannomyces* were negatively correlated with these carbohydrate metabolites.

### Effects of fermented soybean on metabolites of tea new shoots

To reveal the metabolic changes of tea new shoots under fermented soybean fertilization, we screened the differential metabolites of tea shoots by UPLC-MS. Firstly, the OPLS-DA analysis was used to maximize the discrimination among EFS treatment and other treatments, including CK vs EFS, UF vs EFS and NFS vs EFS (Supplementary Figure 4A). The metabolite profiles indicated that enzymatic soybean fertilization was discriminated from control, urea fertilization. Compared with CK, 111 metabolites were identified including 79 up-regulated and 32 down-regulated metabolites. A total of 79 up-regulated metabolites were from lipids, amino acids and their derivatives, phenolic acids, tannins and terpenoids (Supplementary Figure 5A). Compared with UF, 149 metabolites were identified including 77 up-regulated and 72 down-regulated metabolites. There were 77 up-regulated metabolites from tannins, lipids and terpenoids (Supplementary Figure 5B). Compared with NFS, 22 metabolites were identified including 13 up-regulated and 9 down-regulated metabolites (Supplementary Figure 5C). 13 up-regulated metabolites mainly existed in lipids [PE (18:3/18:3 + O3), PE (oxo-11:0/16:0) and 1-Linoleoylglycerol-3-O-glucoside], phenolic acids (cinnamic acid, feruloyl syringic acid and Salicylic acid), Terpenoids (ursolic acid) and flavonoids (galangin-7-O-glucoside). Among them, lipid metabolites in tea new shoots were focused on in this study.

**FIGURE 6:**
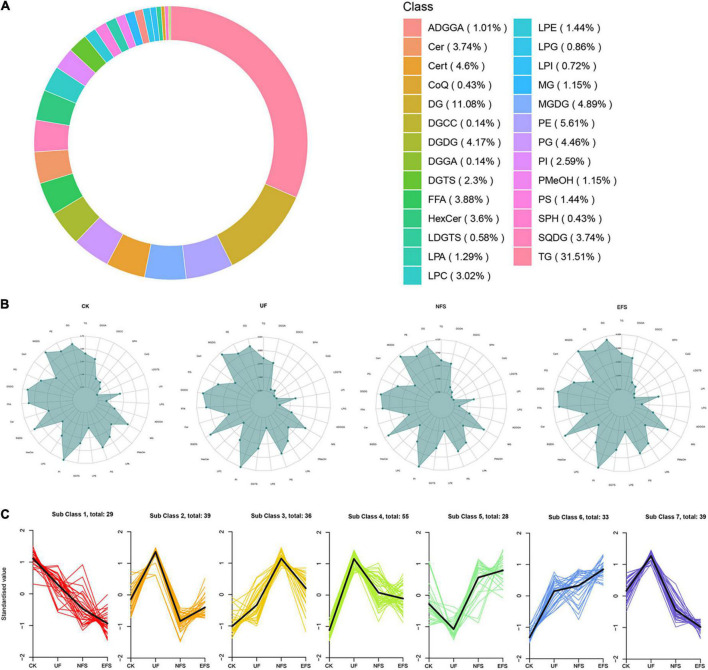
The sub-classes of lipid molecules in tea new shoots. **(A)** The count ring of lipid classes and proportion. **(B)** The radar charts of lipid classes in tea new shoots. **(C)** The K-means cluster analysis of lipid molecules in tea new shoots.

**FIGURE 7:**
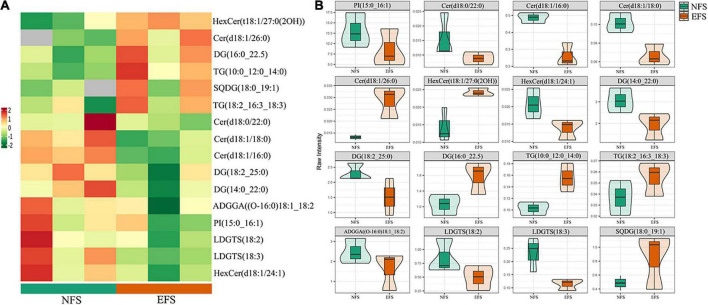
The differential lipid metabolites of tea new shoots in NFS vs EFS group. **(A)** The heatmap of differential metabolites in tea new shoots. **(B)** The violin plot of differential metabolites in tea new shoots.

The OPLS-DA analysis was used to explore the clustering of CK vs EFS, UF vs EFS and NFS vs EFS groups (Supplementary Figure 4B). A total of 695 lipid species were identified, spanning five major lipid categories including 27 fatty acids (FA), 157 glycerophospholipids (GP), 422 glycerolipids (GL), 86 sphingolipids (SL), and 3 prenol lipids (PR). These lipid compounds can be further attributed to 27 subclasses mainly including 219 triacylglycerols (TG), 77 diacylglycerols (DG), 39 phosphatidylethanolamines (PE), 34 monogalactosyldiacylglycerols (MGDG), 32 ceramides (Cert), 31 phosphatidylglycerols (PG), 29 digalactosyldiacylglycerols (DGDG), 27 free fatty acids, 26 cer, 26 sulfoquinovosyl diacylglycerols (SQDG), 25 HexCer and 21 lysophosphatidylcholines (LPC; Figure 6A). The radar charts showed that the proportions of lipid metabolites in 27 categories were different in CK, UF, NFS and EFS (Figure 6B). Lipid metabolites in tea new shoots under different fertilizations were distributed into 7 clusters by using k-means cluster analysis (Figure 6C). The contents of lipid in cluster 3, 5, and 6 of NFS and EFS were higher than in CK and UF. Especially in clusters 5 and 6, the number of lipid metabolites in EFS was more than that in NFS. In cluster 5, 28 lipid metabolites were found including 32% DG, 18% HexCer, 14% TG, 14% LPE and 11% LPC. In cluster 6, 33 lipid metabolites were found including 32% DG, 24% TG, 9% LPC and 9% ADGGA. To compare the advantages of enzymatic fermented soybean fertilization, we carried out an in-depth analysis of the NFS vs EFS group. Lipid metabolites with significant differences were selected using heatmaps and violin plot (Figure 7). 6 lipid metabolites exhibited significant up-regulation in EFS including HexCer [t18:1/27:0(2OH)], Cer (d18:1/26:0), DG (16:0_22:5), TG (10:0_12:0_14:0), TG (18:2_16:3_18:3) and SQDG (18:0_19:1), which focused on glycerolipids and ceramides (Figure 7A). The contents of these metabolites were shown in Figure 7B.

**FIGURE 8:**
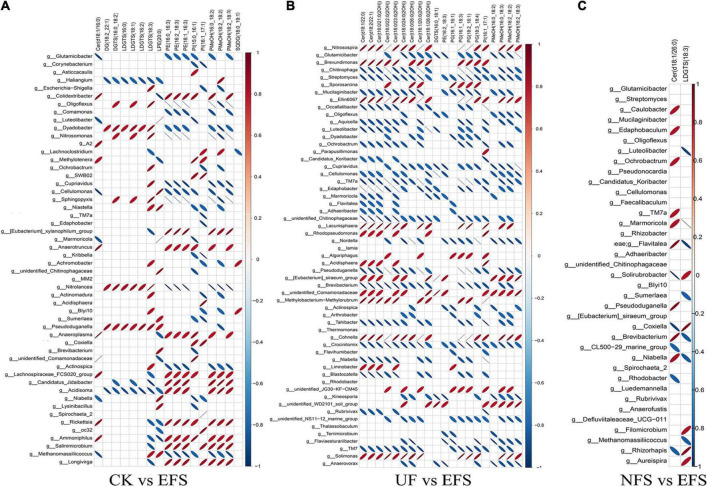
The ellipse heatmap of relationships between bacterial communities and metabolites in tea new shoots. **(A)** The relationship of bacterial communities and metabolites in CK vs EFS group. **(B)** The relationship of bacterial communities and metabolites in UF vs EFS group. **(C)** The relationship of bacterial communities and metabolites in NFS vs EFS group.

**FIGURE 9:**
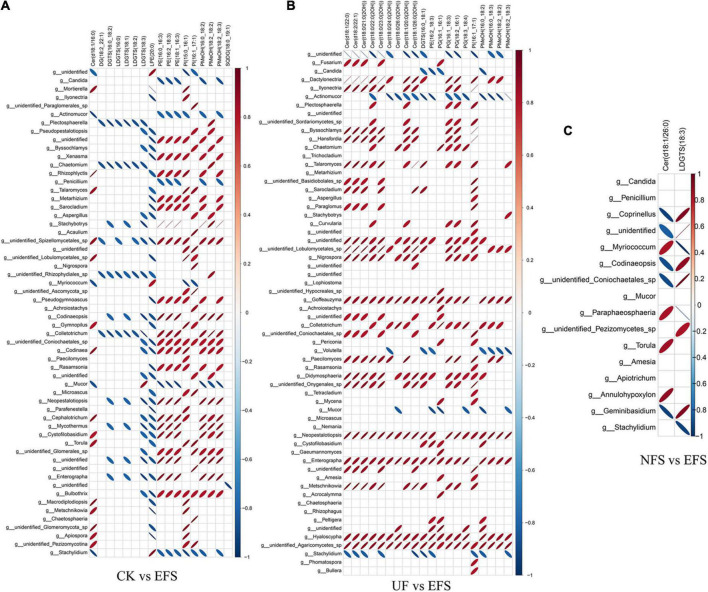
The ellipse heatmap of relationships between soil fungal communities and metabolites in tea new shoots. **(A)** The relationship of fungal communities and metabolites in CK vs EFS group. **(B)** The relationship of fungal communities and metabolites in UF vs EFS group. **(C)** The relationship of fungal communities and metabolites in NFS vs EFS group.

To further explore the relationships between soil microbial communities and metabolites in tea new shoot, we also analyzed their correlations using the Spearman coefficient (Figures 8, 9). In CK vs EFS group, glycerolipids (DG, DGTS and LDGTS) and glycerophospholipids (PE, PI and PMeOH) closely interacted with soil bacterial communities (Figure 8A). DG(18:2_22:1), DGTS(16:0_18:2) and LDGTS[(16:0, 18:1, 18:2, 18:3)] were positively correlated with *Dyadobacter, Nitrolancea* and *Pseudoduganella*, while these metabolites were negatively correlated with *Haliangium* and *Acidisoma.* PE[(16:0_16:3), (16:2_18:3) and (18:1_16:3)], PI[(15:0_16:1) and (16:1_17:1)] and PMeOH[(16:0_18:2), (18:2_18:2) and (18:2_18:3)] were positively correlated with *Colidextribacter, Anaeroplasma, Acidisoma, Rickettsia, Salinimicrobium* and *Longivirga*, while these metabolites were negatively correlated with *Glutamicibacter, Cellulomonas, Sphingopyxis, Nitrolancea, Actinospica* and *Methanomassiliicoccus*. In UF vs EFS group, sphingolipids (Cer) and glycerophospholipids (PG and PMeOH) closely interacted with soil bacterial communities (Figure 8B). Cer [(d18:1/22:0), (d18:2/22:1), (t18:0/21:0(2OH)), (t18:0/22:0(2OH)), (t18:0/23:0(2OH)), (t18:0/24:0(2OH)), (t18:1/20:0(2OH)) and (t18:1/26:0(2OH))] were positively correlated with *Nitrosospira, Brevundimonas, Ellin6067, Lacunisphaera, Cohnella* and *Solimonas*, while these metabolites were negatively correlated with *Chitinophaga, Streptomyces, Mucilaginibacter, Ochrobactrum, TM7a, Edaphobacter, Pseudoduganella, Brevibacterium, Crocinitomix* and *TM7.* PG[(16:1_16:1), (16:1_18:3), (18:2_16:1) and (18:3_18:4)] and PMeOH[(16:0_18:2), (16:0_18:3), (18:2_18:2) and (18:2_18:3) were positively correlated with *Nitrosospira, Cohnella, Lacunisphaera* and *Solimonas*, while these metabolites were negatively correlated with *Glutamicibacter, Chitinophaga, Streptomyces, Nordella, Pseudoduganella, Brevibacterium and Crocinitomix.* In NFS vs EFS group, Cer(d18:1/26:0) and LDGTS (18:3) closely interacted with soil bacterial communities (Figure 8C). Cer(d18:1/26:0) was positively correlated with *Caulobacter, Flavitalea, Pseudoduganella* and *Brevibacterium.* LDGTS (18:3) was positively correlated with *Coxiella* and *Rhizorhapis*. In fungal communities, DG, DGTS and LDGTS were negatively correlated with *Plectosphaerella, Chaetomium*, and *Colletotrichum* in CK vs EFS group. PE, PI and PMeOH were positively correlated with *Srachybotrys, Codinaeopsis, Collectotrichum, Neopestalotiopsis, Cephalotrichum* and *Enterographa* in CK vs EFS group (Figure 9A). In UF vs EFS group, Cer and PG were positively correlated with *Dactylonetria, llyonectria, Nigrospora, Goffeauzyma, Neopestalotiopsis, Enterographa* and *Hyaloscypha* (Figure 9B). In NFS vs EFS group, Cer(d18:1/26:0) and LDGTS(18:3) closely interacted with soil fungal communities (Figure 9C). Cer(d18:1/26:0) was positively correlated with *Annulohypoxylon, Torula* and *Paraphaeosphaeria*. LDGTS (18:3) was positively correlated with *Coprinellus* and *Geminibasidium*.

## Discussion

Soybean nutritional contents, microbial communities and metabolites changed during the fermentation process. The pH value was considered as an important indicator for reflecting the fermentation process and microbial activity (Jiang et al., 2020). In the soybean silage fermentation, all treatments showed lower pH values than the control treatment, and the addition of lactic acid bacteria and molasses led to the highest pH drop (Ni et al., 2017). In the sainfoin silage fermentation, wilted sainfoin and inoculated sainfoin all had low pH and high concentration of lactic acid (Dongmei et al., 2020). Our results also showed that the pH of enzymatic fermented soybean was significantly lower than that of naturally fermented soybean, indicating that the addition of enzymes and bacteria could provide a slightly acidic environment for soybean fermentation (Supplementary Figure 1). It was more conducive to the decomposition of macromolecular nutrients and the improvement of the nutritional value of fermented soybean (Nguyen et al., 2022). These small molecular substances produced by decomposition were beneficial to the growth of tea plants, among which the amino acids produced such as tyrosine, histidine and lysine played positive roles in the growth of tea plants. However, previous studies have not reported the changes of microbial community and metabolite reaction during soybean fermentation, which were easy to be ignored. *Firmicutes, Proteobacteria* and *Bacteroidetes* were the main dominant phyla in different fermentations (Jin et al., 2019; Zhang et al., 2020a), which was consistent with our results. Our results also showed that *Enterobacter* and *Bacillus* were significantly enriched in enzymatic fermented soybean (Supplementary Figure 2). *Bacillus* was the key contributor to degrading organic components in the process of fertilizer fermentation, especially lignocellulosic biomass. *Enterobacter* could excrete acetoin, which was an important physiological metabolite and a quality indicator of fermented products (Xiao and Ping, 2007; Chen et al., 2010). Metabolic differences in soybean could be triggered by bacterial communities in different soybean fermentation processes. During the fermentation of soybean, microbial hydrolases were released and then the complex macromolecules of soybean were degraded into monosaccharides, amino acids and short-chain fatty acids such as palmitic acid, 9,12-octadecanoic acid and stearic acid, etc. (Gupta et al., 2018). Fermentation also affected and changed the lipid content, and then the nutritional composition of soybeans including lipid and protein increased in soybean after fermentation (Nguyen et al., 2022). The contents of phospholipids, fatty acids from neutral lipids and free fatty acids significantly increased after soy sauce fermentation (Zou et al., 2019). In our study, lipid metabolites in EFS were focused on and analyzed to explore the advantages of enzymatic soybean fertilization. The results showed that lipid metabolites accounted for a large proportion of the up-regulated metabolites in soybeans with enzymatic fermentation, such as palmitic acid, oleamide and 11-oxohexadecanoic acid. The obtained findings have demonstrated the role and impact of enzymes and bacteria addition on lipid metabolites formed during soybean fermentation. Therefore, enzymes and bacteria jointly promoted the transformation of macromolecules and increased fermentation efficiency to rapid application in tea plantations.

Enzymatic soybean fertilization improved soil nutrition and altered soil microbial community. Soil environmental factors played important roles in changing the structure of soil microbial communities. In previous studies, soil pH, AN and OM were the key factors altering the microbial communities after organic fertilization (Gu et al., 2019; Zhang et al., 2020b). Our results showed that the contents of TP, AP, and AK significantly increased in soils after enzymatic fermented soybean fertilization, and the activities of S_SC and S_ACP also increased (Tables 1, 2). Due to the tea soil being acidic, phosphorus was easy to combine with iron and aluminum to form insoluble compounds with low mobility. The application of enzymatic fermented soybean could make soil enzymes more active and provide a sufficient material basis for the growth and development of microorganisms. The changes of soil conditions led to the release of nutrients and the inhibition of soil pathogens, which further changed soil microorganisms and soil metabolites. In the present study, *Pseudomonas* was the main species in tea soil, and the abundances of *Glutamicibacter* and *Streptomyces* were higher in soil with enzymatic fermented soybean fertilization. *Pseudomonas* bacteria functioned as a keystone group in soils to mediate complex microbiome-plant feedback and had positive effects on multiple plant growth and phytohormone production through hormonal signaling (Jousset et al., 2017; Finkel et al., 2020; Zhuang et al., 2020). *Glutamicibacter* could tolerate a wide pH range and take a variety of organic substances as carbon sources and showed multiple potential plant growth-promoting traits, including nitrogen fixation and phosphorus dissolution (Feng et al., 2017; Sheng et al., 2018). The functions of *Streptomyces* mainly focused on improving the absorption of nutrients by plants, promoting plant growth, and improving the ability of plants to deal with biotic and abiotic stresses (Jacob et al., 2018; Wu et al., 2018). The results showed that the dominant bacterial communities in tea soil were different from that in fermented soybean fertilizer, indicating that the beneficial microbes in fertilizer did not directly participate in soil improvement and tea growth, but these microbes were guided to promote and activate the resident plant growth-promoting microbes already existing in the rhizosphere of tea plants. In addition, the enrichment of soil nutrients caused substantial shifts in both primary and secondary metabolism within the microbial community, leading to changes in soil functioning (Brown et al., 2022). In this study, lipid (9,12-Octadecadienoic acid), carbohydrate (D-Mannitol and D-Sorbitol) and acid substances were significantly up-regulated in soil with fermented soybean (Figure 3). Carbohydrates were also key metabolites in soil microorganisms, functioning as metabolic substrates and structural cell components (Reardon et al., 2018). D-Mannitol and D-Sorbitol could serve as carbohydrate reserves to store power, transfer compounds, and enhance the growth of plants, fungi and bacteria under stress. A higher concentration of sorbitol could increase microbial diversity and the addition of mannitol could enhance some enzyme activities (Yu et al., 2016). Therefore, fermented soybean fertilization was conducive to shaping an active network of nutrients, microorganisms and metabolites in the soil, which could provide a good soil environment and rich material basis for the growth of tea plants.

Enzymatic soybean fertilization affected the lipid metabolism of tea new shoots, which was closely related to soil microorganisms. Microorganisms were essential for plant growth and had potential benefits in plant secondary metabolism and stress resistance such as promoting photosynthesis, osmotic regulation and antioxidant enzymatic activities (Gabriel et al., 2017; Xiong et al., 2020). As important biomolecules, lipids were represented as precursors of tea aroma and contributed to the tea sensory quality. They could produce many volatile compounds through oxidation and degradation (Ho et al., 2015). Previous studies were focused on lipid components and changes according to different processing procedures of tea (Li et al., 2017, 2019, 2021). However, the lipid profile in tea depends not only on the manufacturing process but also on the fertilizer management in tea plantations. In the present study, glycerolipids accounted for the largest proportion in tea new shoots (Figure 6). In differential metabolites, glycerolipids and glycerophospholipids significantly increased in EFS (Figure 7). Glycerolipids made up a large proportion of plant lipids. Glycerolipids and triacylglycerols in the endoplasmic reticulum could be assembled into fatty acids. The composition of glycerolipids was strongly influenced by plant nutrients. In the model plant system *Arabidopsis*, lipid biosynthesis was significantly affected by nitrogen nutrition, while lipid remodeling was regulated by phosphorus starvation (Kong et al., 2013; Pant et al., 2015). Our results also showed that glycolipids levels positively correlated with some soil bacteria, indicating changes in soil nutrients and bacterial communities jointly influence lipid changes in tea new shoots. In addition, ceramide [mainly Cer (d18:1/26:0)] closely interacted with soil microbial communities in comparison with the naturally fermented soybean fertilization in enzymatic fermented soybean fertilization. Sphingolipids were not only bio-active components of cells with signal transduction function (Worrall et al., 2008), but also the second messenger of plant defense mechanisms participating in various plant stress responses (Markham et al., 2013; Michaelson et al., 2016). Ceramide was the key intermediate of the sphingolipid metabolism pathway, and its synthesis was the starting point of biosynthesis in various complex sphingolipid compounds (Hou et al., 2016). It was proved that sphingolipids had been shown in other crops to be conducive to establishing a mutually beneficial symbiotic relationship between plants and fungi (Fabienne et al., 2011). It also might exist this special beneficial relationship in tea plants according to our results, which could induce the defense response of tea plants and improve the adaptability and disease resistance of tea plants. Further research should focus on the relationships between soil microbiomes and tea lipid metabolites using rhizosphere environment, root secretions and endophytes, etc.

## Conclusion

Our results highlighted the effects of fermented soybean application on soil nutrients, soil microbial communities, soil metabolites, and metabolites in tea new shoots. Compared with urea fertilization, fermented soybean significantly increased the contents of TP, AP, and AK, which were also important environmental factors affecting soil microbial community in tea plantation. Moreover, soil microbial communities had close relationships with soil metabolites and tea new shoots metabolites after enzymatic fermented soybean fertilization. It provides technical support for the rational use of fermented soybean and is of great significance to reduce the amount of chemical fertilizer to protect the soil ecological environment in the tea plantation.

## Data availability statement

The datasets presented in this study can be found in online repositories. The names of the repository and accession numbers can be found below: https://www.ncbi.nlm.nih.gov/, PRJNA863478, PRJNA863595, and PRJNA863609.

## Author contributions

SZ carried out the experiment, collected and analyzed the data, and wrote the manuscript. ZD, LS, and YW raised the hypothesis, designed the experiment, and directed the study. YSh and YSo participated in material preparation and collected sample data. KF directed the laboratory work. RZ, YL, LW, and CB guided and popularized the fertilization technology. All authors contributed to the study and approved the final version.

## Footnotes

1
https://www.metware.cn

2
http://www.anpel.com.cn/

3
http://circos.ca/

## Supplementary material

Supplementary Figure 1 Nutrition analysis of soybean during the fermentation process. **(A)** The changes of physicochemical properties in fermented soybean. **(B)** The contents of 20 amino acids in fermented soybean.

Supplementary Figure 2 The composition of bacterial communities in fermented soybean. **(A)** The histogram of bacterial composition in fermented soybean at the phylum level. **(B)** The histogram of bacterial composition in fermented soybean at the genus level. **(C)** The LDA Effect Size algorithm of bacterial communities with significant differences in fermented soybean.

Supplementary Figure 3 The differential lipid molecules of fermented soybean. **(A)** The heatmap of differential metabolites in soybeans with two fermentations. **(B)** The relationships of bacterial communities and lipid metabolites in soybeans.

Supplementary Figure 4 The OPLS-DA analysis of tea new shoots metabolites. **(A)** The OPLS-DA analysis of tea new shoots metabolites. **(B)** The OPLS-DA analysis of lipid metabolites in tea new shoots.

Supplementary Figure 5 The differential lipid metabolites of tea new shoots in CK vs EF **(A)**, UF vs EF **(B)** and SF vs EF **(C)** group.
