# Supplementary figures and images for: The application of enzymatic fermented soybean effectively regulates associated microbial communities in tea soil and positively affects lipid metabolites in tea new shoots
Source: Front Microbiol. 2022 Aug 23;13:992823. doi: 10.3389/fmicb.2022.992823 (PMC9445587; doi:10.3389/fmicb.2022.992823)

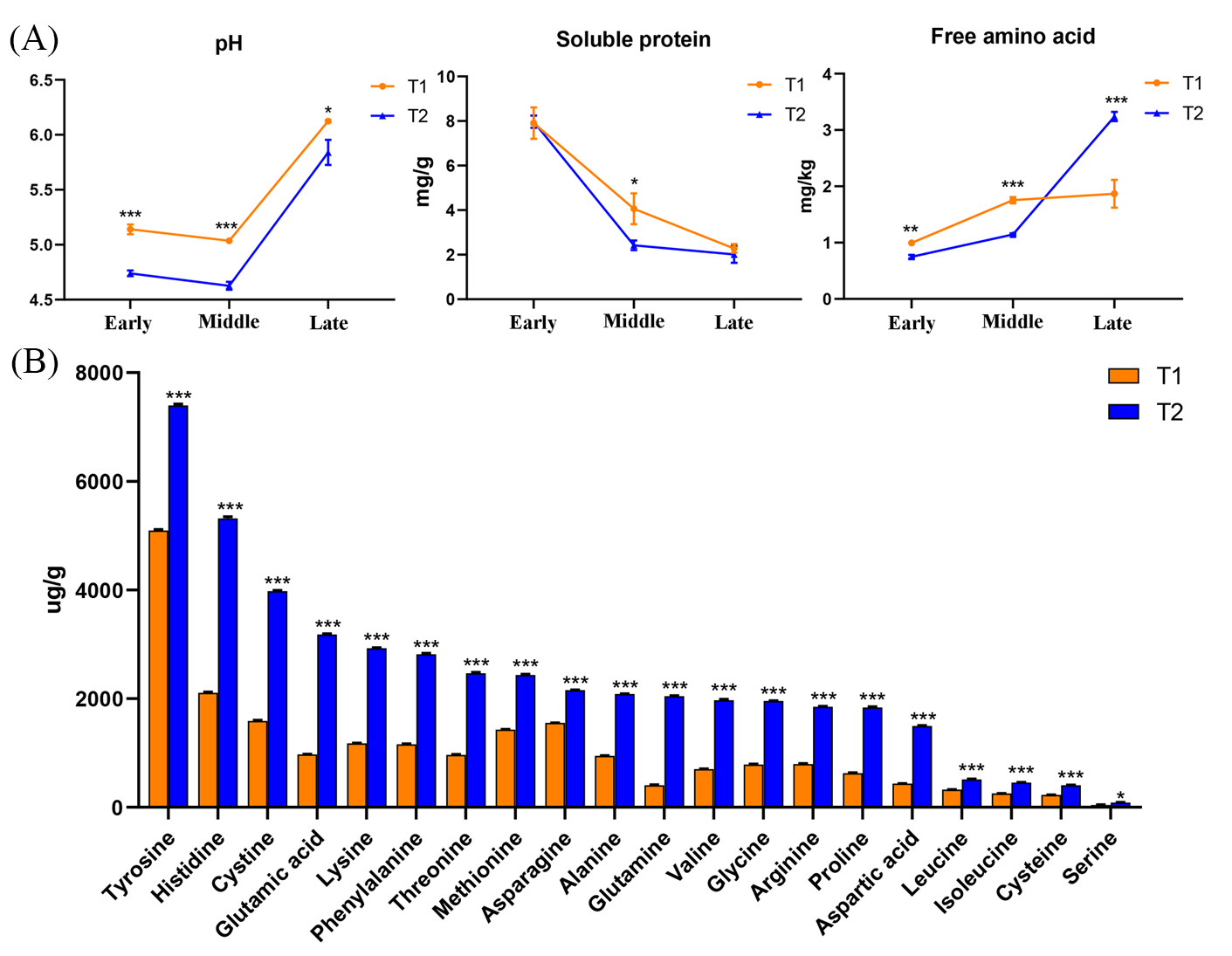

Supplement: Supplementary Figure 1 — Nutrition analysis of soybean during the fermentation process. (A) The changes of physicochemical properties in fermented soybean. (B) The contents of 20 amino acids in fermented soybean. [file Image_1.TIF]

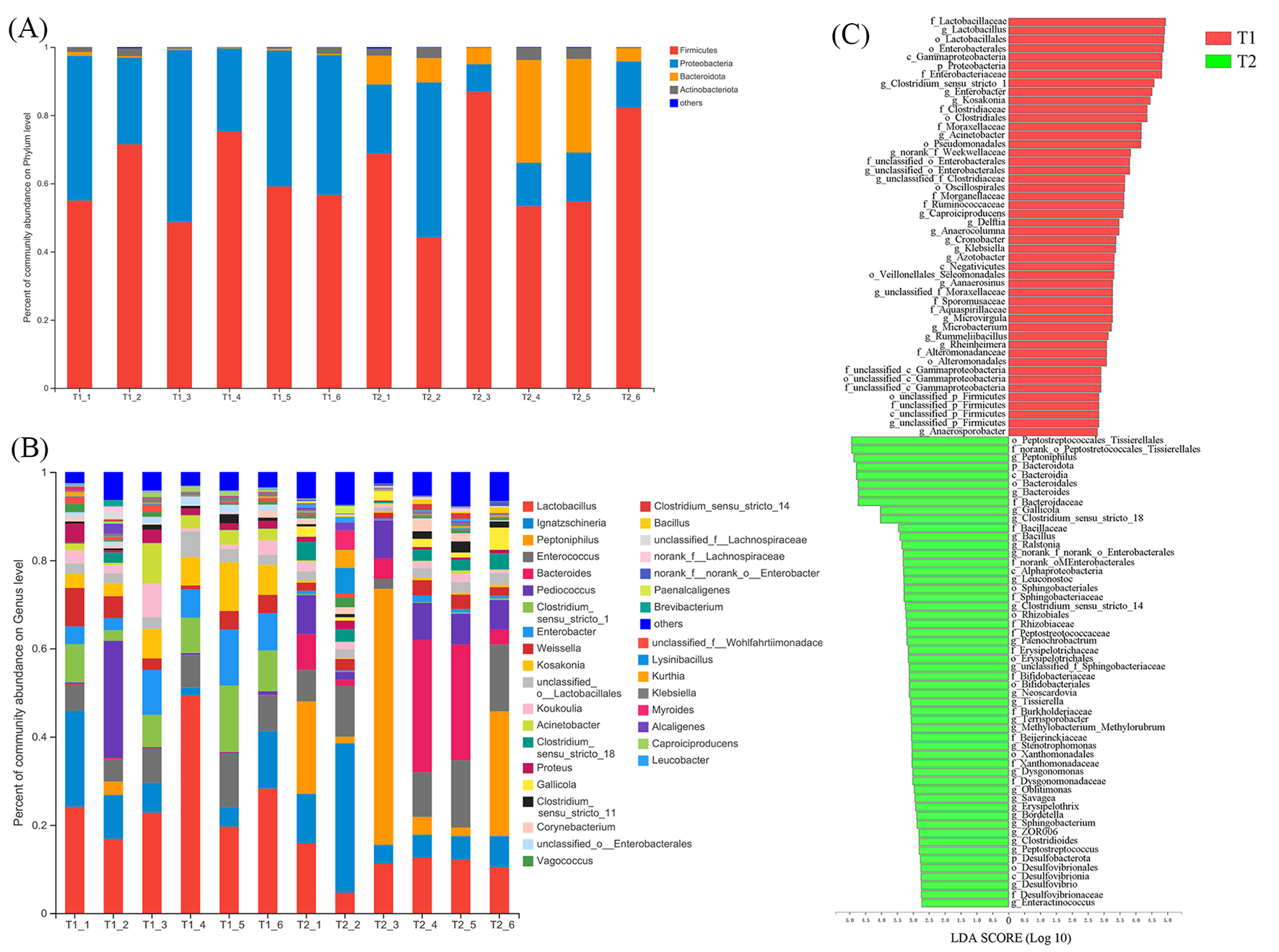

Supplement: Supplementary Figure 2 — The composition of bacterial communities in fermented soybean. (A) The histogram of bacterial composition in fermented soybean at the phylum level. (B) The histogram of bacterial composition in fermented soybean at the genus level. (C) The LDA Effect Size algorithm of bacterial communities with significant differences in fermented soybean. [file Image_2.TIF]

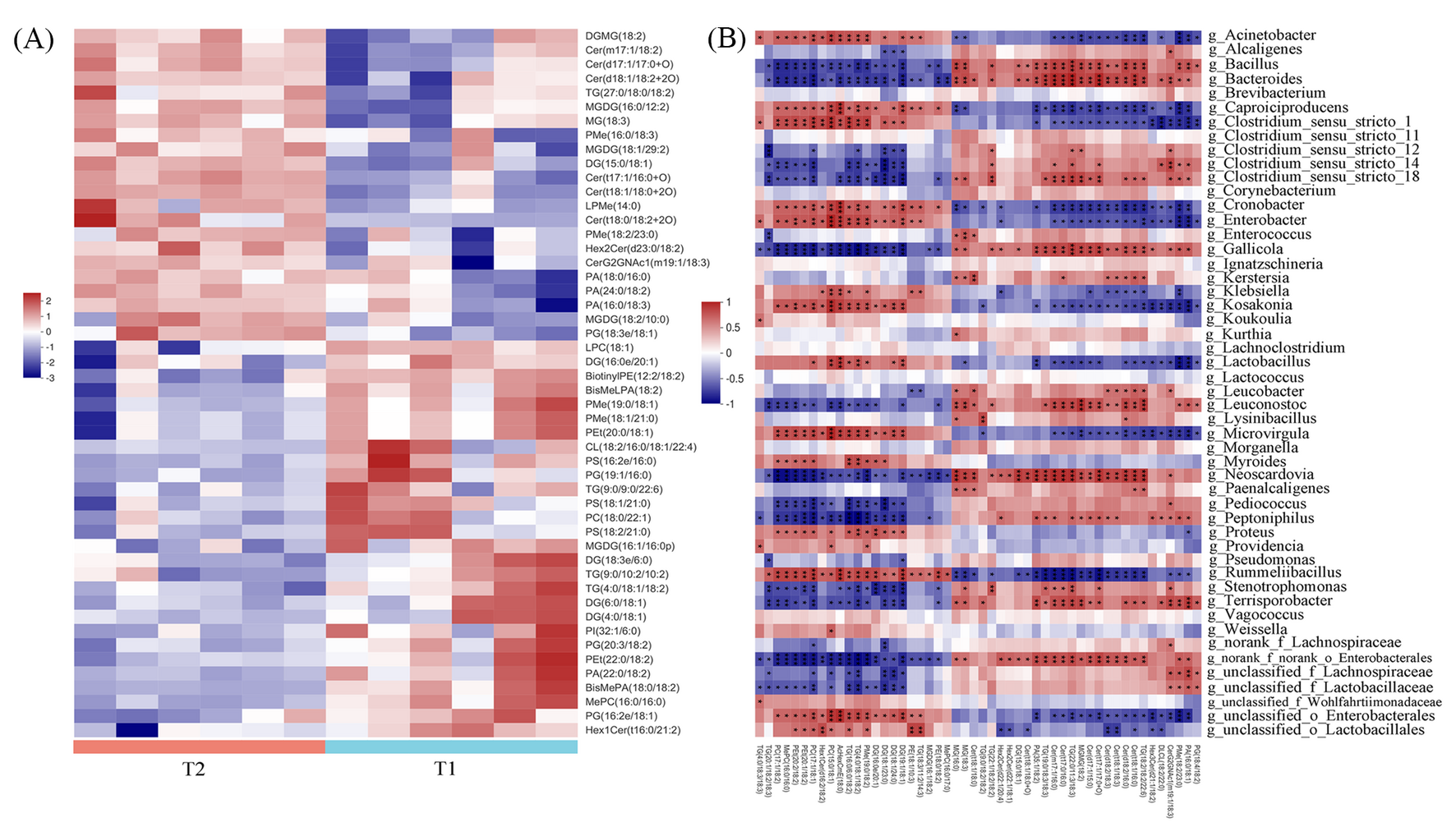

Supplement: Supplementary Figure 3 — The differential lipid molecules of fermented soybean. (A) The heatmap of differential metabolites in soybeans with two fermentations. (B) The relationships of bacterial communities and lipid metabolites in soybeans. [file Image_3.TIF]

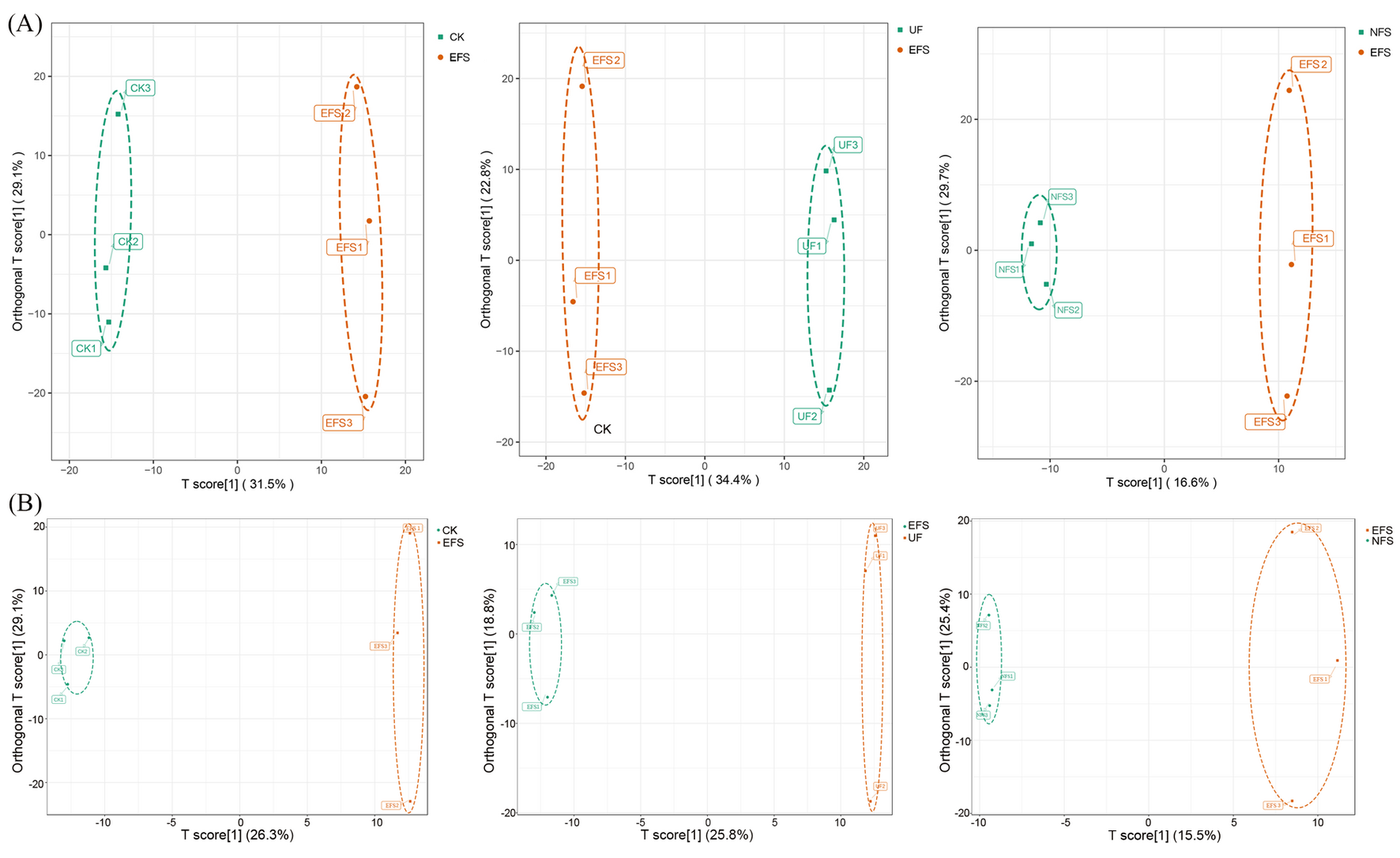

Supplement: Supplementary Figure 4 — The OPLS-DA analysis of tea new shoots metabolites. (A) The OPLS-DA analysis of tea new shoots metabolites. (B) The OPLS-DA analysis of lipid metabolites in tea new shoots. [file Image_4.TIF]

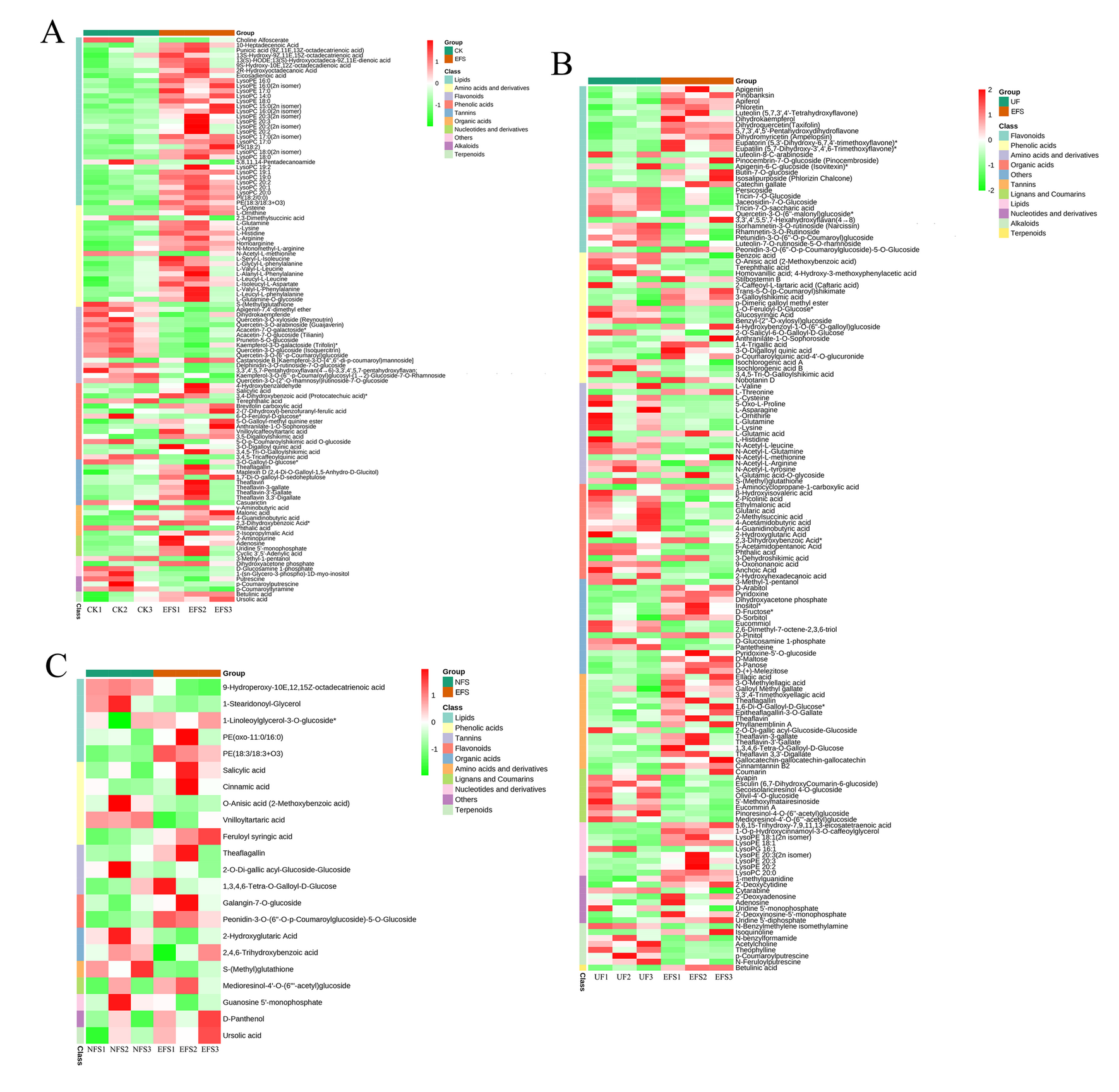

Supplement: Supplementary Figure 5 — The differential lipid metabolites of tea new shoots in CK vs EF (A), UF vs EF (B) and SF vs EF (C) group. [file Image_5.TIF]
